# Atomistic Simulations of the Elastic Compression of Platinum Nanoparticles

**DOI:** 10.1186/s11671-022-03734-z

**Published:** 2022-10-03

**Authors:** Ingrid M. Padilla Espinosa, Tevis D. B. Jacobs, Ashlie Martini

**Affiliations:** 1 Department of Mechanical Engineering, University of California Merced, 5200 Lake Rd, Merced, CA 95340 USA; 2 Department of Mechanical Engineering and Materials Science, University of Pittsburgh, 3700 O’Hara St., Benedum Hall, Room 636, Pittsburgh, PA 15261 USA

**Keywords:** Nanoparticles, Elastic modulus, Size-dependence, Molecular dynamics, Platinum

## Abstract

The elastic behavior of nanoparticles depends strongly on particle shape, size, and crystallographic orientation. Many prior investigations have characterized the elastic modulus of nanoscale particles using experiments or simulations; however their reported values vary widely depending on the methods for measurement and calculation. To understand these discrepancies, we used classical molecular dynamics simulation to model the compression of platinum nanoparticles with two different polyhedral shapes and a range of sizes from 4 to 20 nm, loaded in two different crystal orientations. Multiple standard methods were used to calculate the elastic modulus from stress-vs-strain data for each nanoparticle. The magnitudes and particle-size dependence of the resulting moduli varied with calculation method and, even for larger nanoparticles where bulk-like behavior may be expected, the effective elastic modulus depended strongly on shape and orientation. Analysis of per-atom stress distributions indicated that the shape- and orientation-dependence arise due to stress triaxiality and inhomogeneity across the particle. When the effective elastic modulus was recalculated using a representative volume element in the center of a large nanoparticle, the elastic modulus had the expected value for each orientation and was shape independent. It is only for single-digit nanoparticles that meaningful differences emerged, where even the very center of the particle had a lower modulus due to the effect of the surface. These findings provide better understanding of the elastic properties of nanoparticles and disentangle geometric contributions (such as stress triaxiality and spatial inhomogeneity) from true changes in elastic properties of the nanoscale material.

**Supplementary Information:**

The online version contains supplementary material available at 10.1186/s11671-022-03734-z.

## Introduction

Face-centered cubic (FCC) nanoparticles are widely used in applications such as drug delivery [1, 2], catalysis [3], tribology [4], nanolithography [5], electrochemical sensors [6], and as fillers in polymer composites [7]. Many of these applications apply mechanical loads resulting in compression of the particles, by design or inadvertently during use. However, the elastic response of nanoparticles to compression loading can differ dramatically from the same material in the bulk [8]. At larger length scales, the near-surface material is only a small fraction of the overall volume, so surface effects are negligible and the elastic modulus is an intensive property that depends only on the material. For nanoparticles, however, the surface effects are significant, and the size and shape of particles determine their elastic behavior [8–11]. These effects have been characterized using experiments, theory, and simulations.

The elastic properties of nanoparticles have been measured experimentally using force-displacement curves from compression tests with atomic force microscopy (AFM) or AFM with *in situ* transmission electron microscopy (TEM) [12, 13], and by measuring lattice distance change with pressure using x-ray diffraction (XRD) in anvil cells [14–16]. From AFM and *in situ* TEM measurements, the elastic modulus is typically calculated using Hertz theory for very small contact forces [17, 18]. This calculation requires accurate measurement of the contact area or tip displacement and so is limited by the resolution of the instrument [17, 19]. XRD techniques require a very high spatial resolution to measure properties and may not be suitable to study nanoparticle properties at the smallest scales [14]. Experiments are also limited in their ability to explain the origins of nanoparticle properties because they cannot identify surface- and bulk-material contributions to measured elasticity.

Theoretical models of the elastic properties of nanostructures are based on the difference between surface and bulk properties [10, 20]. This difference has been shown to depend on the geometry of the nanostructure, its size, and a parameter that relates surface elastic constants to bulk elastic constants [20]. To characterize nanoparticle elasticity by continuum field theories, the particles are described as bodies composed of bulk material and a bounding surface. The bulk properties are inherent to the material, and the bounding surface properties are defined from the surface stress tensor [21, 22]. The bounding surface mathematically represents the atomic surface and a few layers below the surface [20]. At and near the surface, the atoms are under-coordinated and relax by shifting inward toward the bulk, but their positions are also constrained by interactions with neighboring surface atoms, resulting in tensile surface stress without external force [23]. Although theoretical approaches describe the differences between surface and bulk properties of nanoparticles, the calculation of surface elastic properties requires additional molecular models and analysis [20, 23, 24]. Furthermore, most theoretical models have been developed for curved surfaces like spheres and cylinders, which may not reflect the faceted polyhedral shapes formed by small nanoparticles [23, 25].

Elastic properties of nanoparticles have been computed using meso-scale finite element methods (FEM) [12, 25] and atomic-scale molecular dynamics (MD) simulation [29–31]. FEM approaches can model stress distributions for different shapes that resemble nanoparticles while requiring fewer computational resources than atomic-scale simulation methods [25]. However, FEM cannot provide atomic resolution and is based on continuum assumptions that may not hold true at the surface of nanoscale structures [26].

Alternatively, MD simulations provide atomistic models of the nanoparticles under compression by a virtual or explicit indenter and also enable modeling of nanoparticles with different sizes and shapes. A limitation of MD-based approaches for calculating elastic modulus is related to the definition of stress. Two main approaches have been used to calculate stress from MD models of compression [26]. The first approach defines stress as the force on the indenter divided by the contact area. However, area of contact is poorly defined at the nanoscale and a range of values can be obtained depending on how it is calculated [27]. Often contact area is calculated by enclosing the layer of atoms adjacent to the compressing indenter by a convex hull [28–31]. An alternative is to use the area of the nanoparticle at its mid-height, which is well defined in a simulation and is commonly used in mechanical tests [29], but represents a lower, average value of stress and cannot capture the higher stresses that exist near the indenter contact. The second approach is based on the concept of virial stress. [31–33]. In this approach, the stress tensor for an atom is derived from an expression that includes both kinetic energy and potential energy due to intra- and inter-molecular interactions [34]. The virial calculation gives a result in units of stress times volume, i.e., energy, and requires estimation of the relevant volume to obtain stress. Volume is often calculated using Voronoï tessellation or Delanauy triangulation [34, 35]. In the virial stress approach, stress is effectively calculated as an average over the volume of the nanoparticle (or the region of analysis) and so might not differentiate the contributions of features such as facets, edges, and corners.

The size dependence of the elastic properties of nanoparticles has been widely studied using experiments, theory, and simulations. Most analytical and numerical models [18, 36] and experimental studies [37, 38] predict that the elastic modulus decreases with decreasing size. MD simulations and theoretical approaches have shown that this size effect can be associated with lower cohesive energy for smaller particles [18, 39, 40]. Nanoparticle size effects can also be understood in the context of grain size in bulk materials. A previous study of the effect of grain size on the elastic modulus of platinum found a linear relationship between modulus and the reciprocal of grain size [41]. This was explained by the fact that atoms at the grain boundaries have a larger potential energy and can move more easily than atoms inside the grain, so these boundary atoms contribute less to the elastic resistance; then the modulus is low for smaller grains in which there is a larger proportion of boundary atoms. However, some theoretical studies [9, 42] have predicted an increase in elastic modulus with decreasing size. This trend was attributed to the increase in bond energy from the reduced bond length at the surface for smaller nanoparticles, which resulted in bond strengthening.

The effect of nanoparticle shape and orientation has also been characterized in previous investigations. Theoretical studies proposed that shape is relevant to elastic properties only for very small nanoparticles (below approximately 20 nm) [40]. For example, a MD study [29] of 15-nm $\mathrm {Ni_{3}Al}$ nanoparticles with sharp and blunt edges showed that the elastic modulus of cubic nanoparticles with sharp edges was less than 10% of the bulk value while, when the edges of the particle were rounded, the elastic modulus increased to about twice the bulk elastic modulus [29]. Similarly, a MD study of silicon nanoparticles with spherical, cubic, cubic-with-blunt edges, and Wulff-like shapes showed that only perfect cube structures had an elastic modulus equivalent to the bulk value, while different shapes had moduli up to three times the bulk value [31]. FEM calculations of polyhedral nanoparticles with different ratios of {111} and {100} facets have shown that the elastic modulus decreases with an increase of {100} facets [25]. Atomistic simulations on nanoscale films of various materials showed that the elastic properties varied with crystallographic orientation [43]. The effect of the loading direction relative to crystallographic orientation on the elastic properties of nanoparticles has also been observed in finite element analyses [25]. The calculated force-distance curves were fit with Hertzian models, and different constants were calculated and proposed to predict the elastic modulus of polyhedral nanoparticles based on the ratios of {111} and {100} facets and the loading orientation. Thus, for single-crystal nanoparticles, the direction of the loading relative to the crystallographic orientation is expected to affect elasticity.

In summary, it is well established that the elastic response of nanoparticles depends not only on their material, but also on their size, shape, and orientation. However, it remains difficult to disentangle geometric contributions (such as stress triaxiality and spatial inhomogeneity) from true changes in the elastic properties of the nanoscale material. While MD simulations overcome this challenge by explicitly modeling atoms, they are limited by the fact that stress can be calculated in several different ways, some of which are based on assumptions that may not apply to nanoparticles. Also, previous MD-based studies have reported different or even contradictory findings; for example, the elastic modulus has been shown to either increase [18, 36] or decrease [9] with nanoparticle size, as mentioned above. In this context, the purpose of the present study is to, first, compare the most common methods used to calculate modulus to understand the origin of the differences among them and, second, to distinguish between geometric and nanoscale effects. We use MD simulations to model compression of two different platinum nanoparticle shapes with different sizes and crystallographic orientations relative to the loading direction. First, multiple commonly used methods for calculating the elastic modulus of nanoparticles are compared. Then, atomic stress distributions are analyzed to explain deviations from expected trends. Lastly, the elastic behavior of nanoparticles is described based on the triaxial stress of a representative volume element at the center of the nanoparticle in order to understand the size, shape, and orientation dependence.

## Methods

**Fig. 1:**
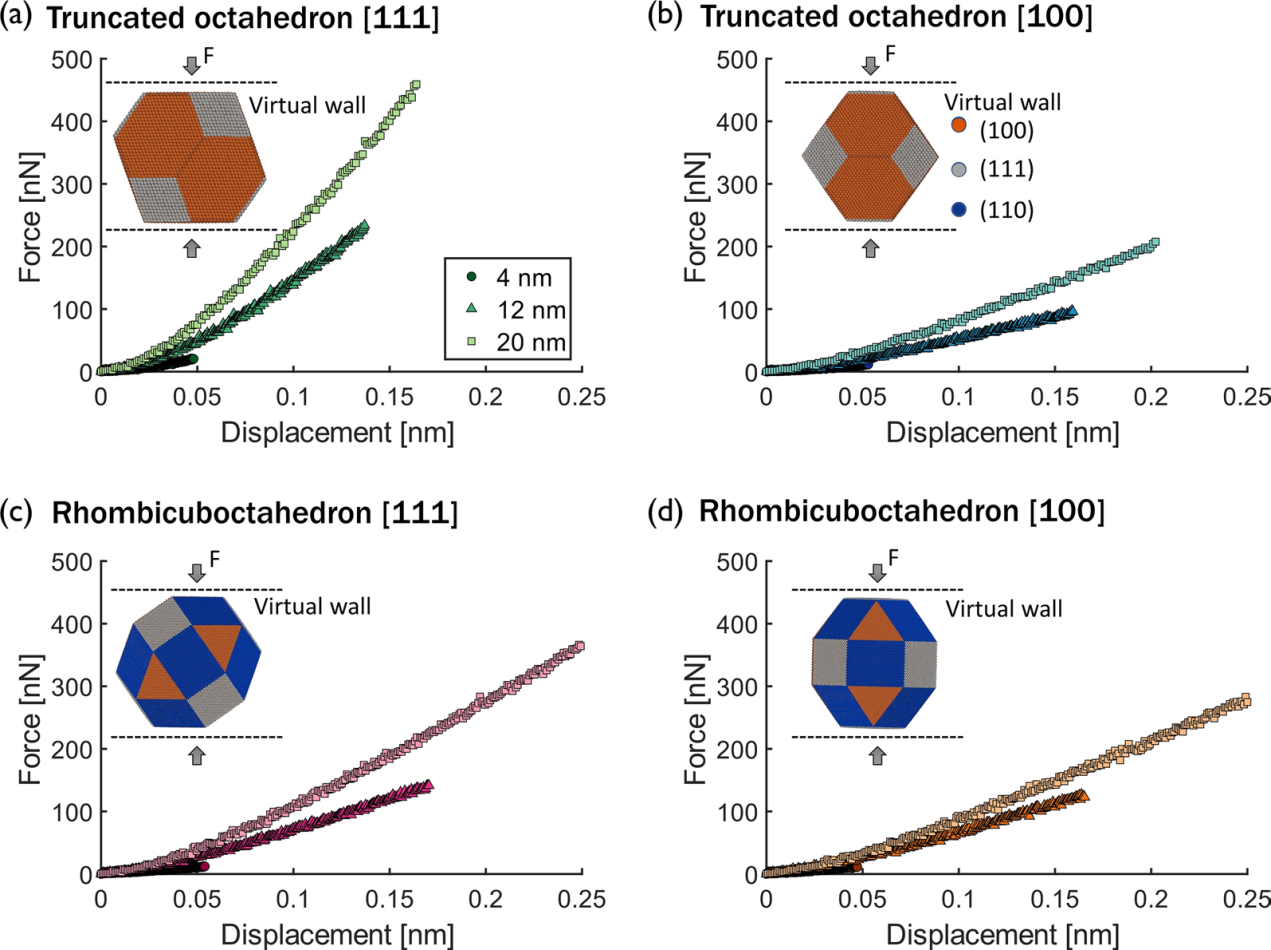
Nanoparticle models and force-vs-displacement compression response. Force vs displacement of nanoparticles with two different shapes,** a**,** b** truncated octahedron and** c**,** d** rhombicuboctahedron. The nanoparticles were tested in two crystallographic orientations with respect to the direction of the load,** a**,** c** corresponding to the {111} orientation and** b**, **d** to the {100} orientation. Insets are snapshots of the models with atom color corresponding to the {100}, {111}, and {110} facets

FCC nanoparticles can be synthesized in many different shapes including icosahedra [44], tetrahedra [45], cuboctahedra or “quasi spherical” [46], cubes [47], and truncated octahedra [48]. Most of these nanoparticle shapes are bound by lowest-energy facets {111} and {100} [49, 50], although a few have facets bound by {110} planes and higher-energy surfaces. Here, MD models of the elastic compression of truncated octahedron and rhombicuboctahedron platinum nanoparticles between 4 nm and 20 nm in diameter were developed. These shapes were selected because they are likely to occur in platinum [51] and because their different facets (truncated octahedron bound by {100} and {111} facets, rhombicuboctahedron by {100}, {111}, and {110} facets) enable analysis of the effect of facet orientation on elastic behavior. The models are shown in the insets of Fig. 1.

The nanoparticles were created using LAMMPS (Large-scale Atomic/Molecular Massively Parallel Simulator) [52] and OVITO software [53]. The nanoparticles were single crystals “carved” from a large FCC block of atoms with the lattice parameter of platinum. For each particle, the size was defined as the minimum diameter of a sphere that enclosed the entire nanoparticle shape. All simulations were performed with the LAMMPS package. The embedded atom method (EAM) [54] potential was used to describe the atomic interactions, based on our previous study that showed that this potential could accurately reproduce the mechanical and surface properties of bulk platinum, and accurately predict platinum nanoparticle stability [55]. The nanoparticles were initially geometrically optimized through an energy minimization process, using the conjugate gradient method until the difference in relative energy between iterations divided by the average energy was less than 1x10^-7^. Next, the velocity was randomized following a Boltzmann distribution at a temperature equal to 300 K. Then, the nanoparticles were equilibrated at 300 K for 200 ps using a canonical ensemble with a Langevin thermostat and a damping parameter of 100 fs. A time step of 2 fs was used for all the dynamics.

Following the equilibration process, the particles were compressed using two parallel virtual walls that moved from the top and bottom of the simulation box toward the center of the nanoparticle, as shown in the insets of Fig. 1. The compression was applied in two directions, normal to a {111} facet or normal to a {100} facet, until a maximum strain of 3% was reached. To allow for unconstrained deformation, a gap three times larger than the nanoparticle was introduced between the nanoparticle and the edges of the simulation box, ensuring that the nanoparticles could freely expand in the direction perpendicular to the load. The walls interacted with the nanoparticle atoms following a purely repulsive harmonic potential described by $E={\epsilon (r-r_{c})^2; r<r_{c}}$, where *E* is the energy of wall-particle interaction, $\epsilon$ is the spring constant of the harmonic wall, *r* is the distance from the particle to the wall, and $r_{c}$ is the cutoff distance at which the particle no longer interacts with the wall. Different values of $\epsilon$ and cutoff were used and the results (shown in Additional file 1: Fig. A1) confirmed that the compression response of the particle was independent of these parameters. A spring constant of 8000 N/m and cutoff of 0.2 nm were used for all subsequent simulations. Experimental strain rates are several orders of magnitude smaller than the strain rates used in MD [56]. Strain rate effects have been observed previously in MD simulations of compressed nanomaterials and the lowest strain rate accessible is usually recommended to model nanomaterial deformation that resembles experimental results [56]. Simulations were run at different strain rates for the 6 nm truncated octahedron nanoparticle and it was found that there was no statistically significant effect of strain rate on the load-vs-displacement curve (see Additional file 1: Fig. A2). Based on this analysis, subsequent compression simulations were run at a strain rate of $1\times 10^7~s^{-1}$. The force on the virtual walls and the displacement and virial stress per atom were recorded every 5 ps and averaged over every 2 ps.

## Results and Discussion

The force-displacement curves for three nanoparticle sizes (4 nm, 12 nm, and 20 nm) compressed to 3% strain are shown in Fig. 1 for (a) the truncated octahedron with load perpendicular to a {111} facet, (b) the truncated octahedron with load perpendicular to a {100} facet, (c) the rhombicuboctahedron with load perpendicular to a {111} facet, and (d) the rhombicuboctahedron with load perpendicular to a {100} facet. The force-displacement curves for nanoparticles of different sizes do not have the same rate of increase since larger forces are needed to produce the same displacement in large nanoparticles compared to small nanoparticles. In addition to size, Fig. 1 shows that the nanoparticle force response to compression depends on shape and orientation.

Next, we evaluated the elastic modulus of the nanoparticles. Elastic modulus is an intensive property of bulk materials. However, at the nanoscale, materials are not homogeneous and properties may differ near free surfaces. Therefore, we subsequently refer to the calculated elastic modulus of nanoparticles as the “effective elastic modulus” $\text {E}_{\text {eff}}$.^1^ First, the Hertz theory has been used to obtain the effective elastic modulus of nanoparticles at small strains from experimental indentation and molecular models [17, 26]. Based on the particle geometry and the virtual wall used to mimic an indenter, the force-vs-displacement data was fit to the Hertz equation for a rigid cylinder with flat end and an elastic half-space: $P=2a\text {E}_{\text {eff}}d$, where *P* is load, *a* is radius of contact, and *d* is indentation depth [57, 58]. Second, the virial stress$\times$volume was recorded during the compression process and divided by the nanoparticle volume calculated using Delaunay triangulation at each strain. The calculation was based on the virial stress in the direction of the applied load ($\sigma _{z}$), following the uniaxial approach used in previous nanoparticle simulations [29, 41]. Third, the stress was calculated as the force on the virtual wall divided by the area of contact between the nanoparticle and virtual wall, as done in [31]. Fourth, stress was calculated as force over area, but the area used for the calculation is that of the cross-section at the center of the nanoparticle. The area of contact and the area of the cross-section at the middle of the nanoparticle were approximated using Delaunay triangulation, as done in [29]. In all analyses, the strain was calculated as the ratio of the change in nanoparticle height at a given strain and the pre-strained height of the particle. For the last three approaches, the effective elastic modulus was calculated as the slope of the stress-strain curve. Error bars reflect 95% confidence intervals of the fit.

The effective elastic moduli of the nanoparticles loaded in the {111} and {100} orientations were compared to the directional elastic modulus $\text {E}_{\text {dir}}$ of bulk platinum in the {111} and {100} orientations computed as:

1 $$\begin{aligned} \frac{1}{\text {E}_{\text {dir}}}=S_{11}-2\left[ \left( S_{11}-S_{12} \right) -\frac{1}{2}S_{44} \right] \left( l^{2}m^{2}+m^{2}n^{2}+l^{2}n^{2} \right) \end{aligned}$$

where $S_{ij}$ are the elastic constants and *l*, *m*, *n* are the cosines of the angles between the direction of $\text {E}_{\text {dir}}$ and the crystal axes [59]. The elastic constants for platinum described by the potential used in this study were determined in previous work [55, 60]. The bulk elastic modulus was calculated using Eq. 1 to be 211 GPa for the {111} orientation and 107 GPa for the {100} orientation.

**Fig. 2:**
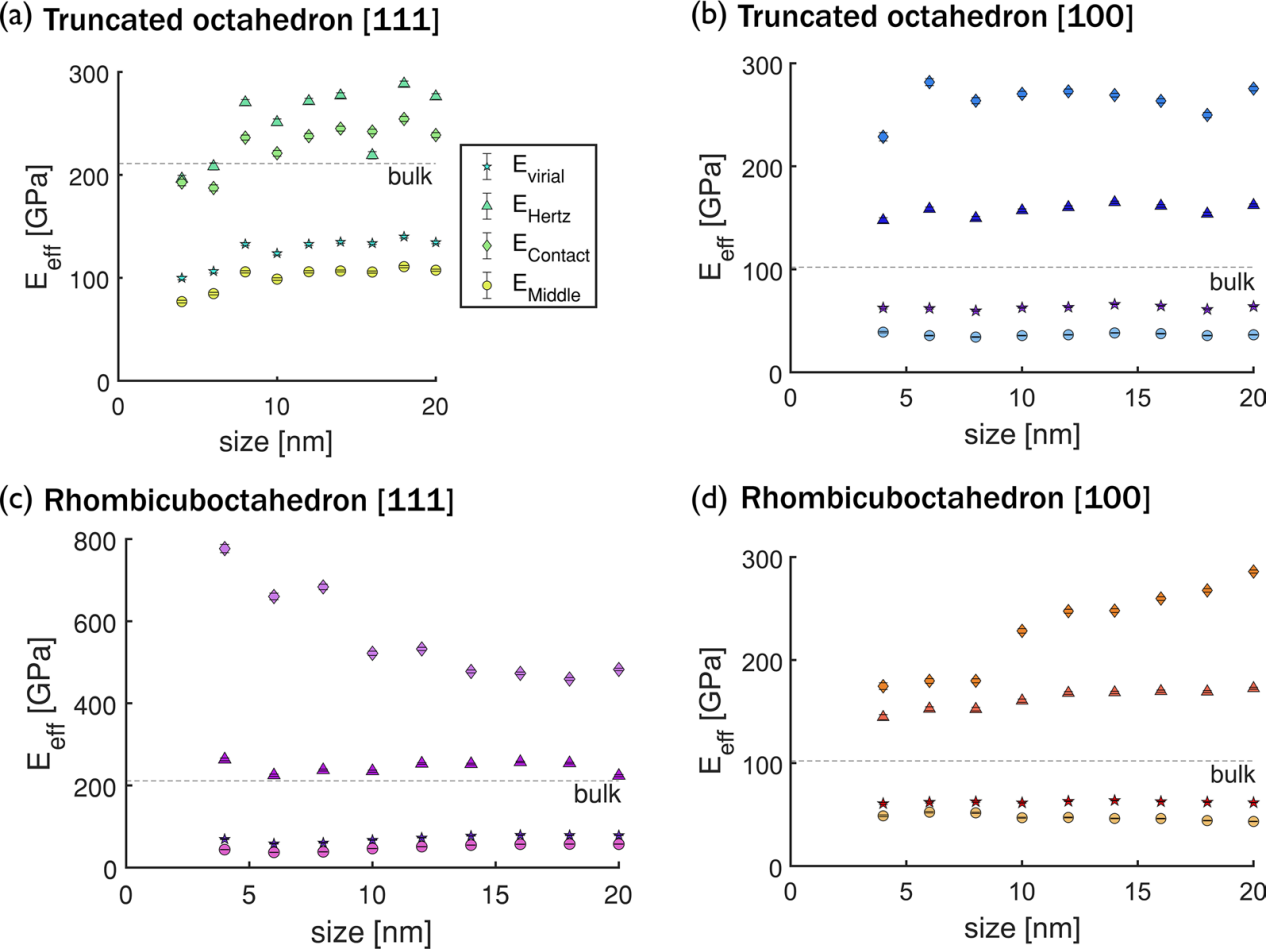
Size dependence of the effective elastic modulus calculated with different approaches. Effective elastic modulus calculated from the virial stress (stars), Hertz approximation of a rigid cylinder and a half space (triangles), force over contact area (diamond), and force over middle area (square) for two nanoparticle shapes, **a** ,**b** truncated octahedron and **c**,** d** rhombicuboctahedron, and two crystallographic orientations,** a**,** c** corresponding to {111} and** b**,** d** to {100} orientation. The bulk value calculated from simulations is shown as a dashed line

Figure  2 shows the effective elastic modulus E$_{\text {eff}}$ of particles from 4 to 20 nm with different shape and orientation combinations, calculated using the four methods described above. The effective elastic modulus of the bulk material in a given orientation is shown as a dashed line in each plot. For the truncated octahedron, size dependence of the effective elastic modulus is observed for the {111} orientation by all methods (Fig. 2a), but this trend is not observed for the {100} orientation (Fig. 2b). Size dependence is also observed for the rhombicuboctahedron when the effective elastic modulus is calculated as force over contact area. However, while the effective elastic modulus decreases with increasing size for the rhombicuboctahedron in the {111} orientation (diamonds in Fig. 2c), the opposite trend is observed for the {100} orientation (diamonds in Fig. 2d).

**Fig. 3:**
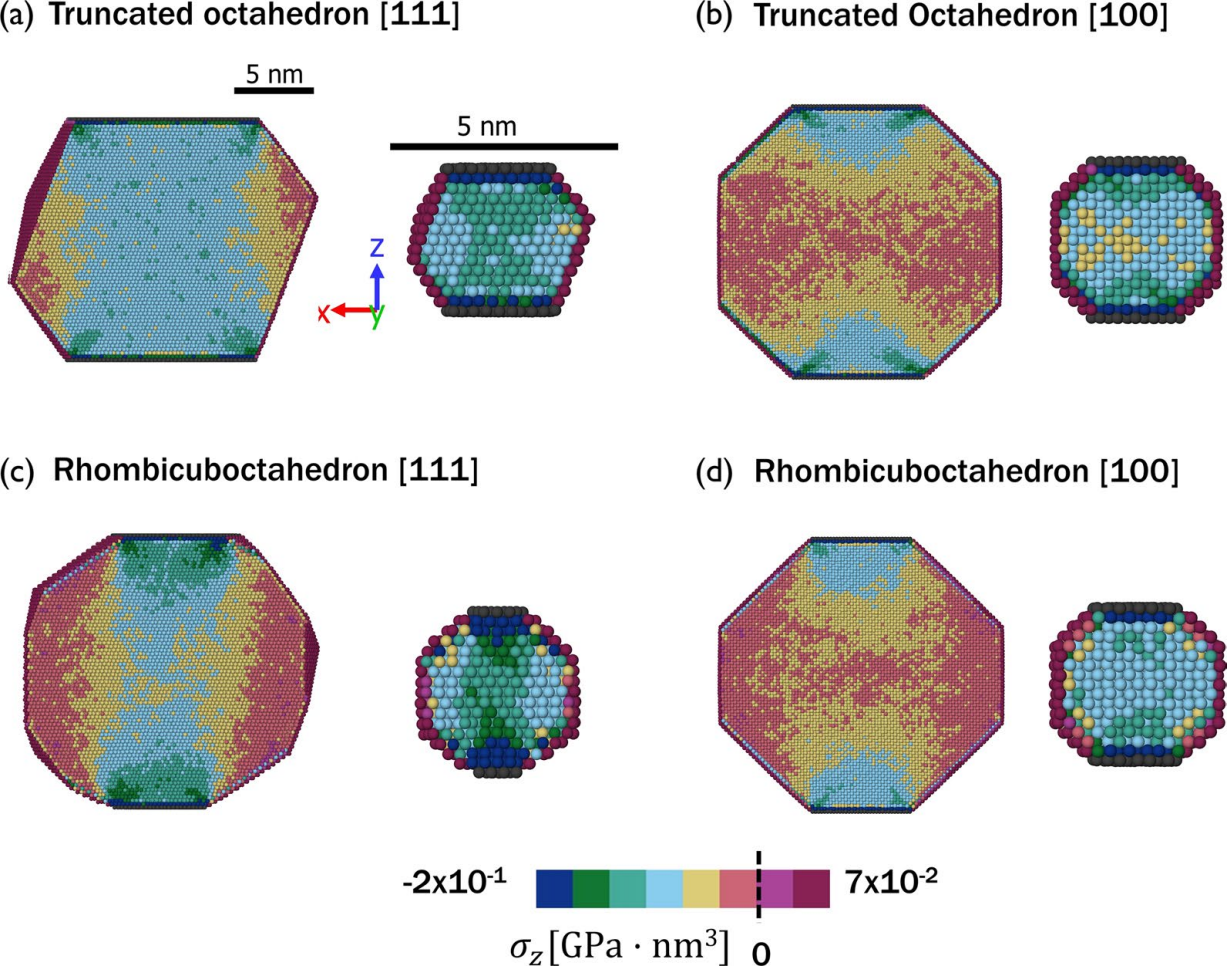
Virial stress distributions for 20 nm and 4 nm nanoparticles. Virial stress on cross-sections parallel to the loading direction for the two nanoparticle shapes,** a**,** b** truncated octahedron and** c**,** d** rhombicuboctahedron, and two crystallographic orientations,** a**,** c** correspond to {111} and** b**,** d** to {100} orientation. The color scale has units of GPa$\cdot$nm^3^

Beyond the differences between these calculation methods in predicted size dependence, the magnitudes of the effective elastic moduli differ in Fig. 2. Theoretically, when a nanoparticle is large enough, the surface effects are negligible and it can be considered as a homogeneous body and the elastic modulus should not be strongly shape dependant [10, 40]. So, an accurate calculation method should predict convergence of the effective elastic modulus to the bulk value for larger particles and be independent of shape for a given crystallographic orientation. The stress calculated as force over contact area overestimates the effective elastic modulus of both orientations. The Hertz approximation provides an accurate prediction for the {111} orientations, but overestimates the effective elastic modulus for {100} orientations. The approximation of stress as force over middle area underestimates the stress and therefore the modulus. The virial approximation underestimates the effective elastic modulus compared to the bulk value for all the nanoparticles and there is a significant difference between the values predicted for the two different shapes, even for larger particles, contrary to expectation.

To understand how nanoparticle size, shape, and orientation affect elastic behavior, the distributions of the virial stress$\times$volume in the direction of the applied load were calculated at 3% strain, averaged over 2 ps. Cross-sections of the nanoparticles as viewed from a direction perpendicular to the loading direction (cross-sectional view defined in Additional file 1: Fig. A3) with the atoms colored by stress magnitude are shown in Fig. 3 for 4-nm and 20-nm nanoparticles. Importantly, the stress is inhomogeneously distributed across the nanoparticles and the stress ranges from compressive to tensile depending on the location within the particle. Tensile stress is observed near the surface and the proportion of these tensile surface regions is much larger for the smaller particles. For all combinations of particle shape and orientation, the compressive stress is largest near the top and bottom of the particles where the cross-sectional area is the smallest. The maximum compressive stress is consistently observed near the corners of the contact areas. However, these figures also show that the stress distributions are very different for the different shapes and orientations.

**Fig. 4:**
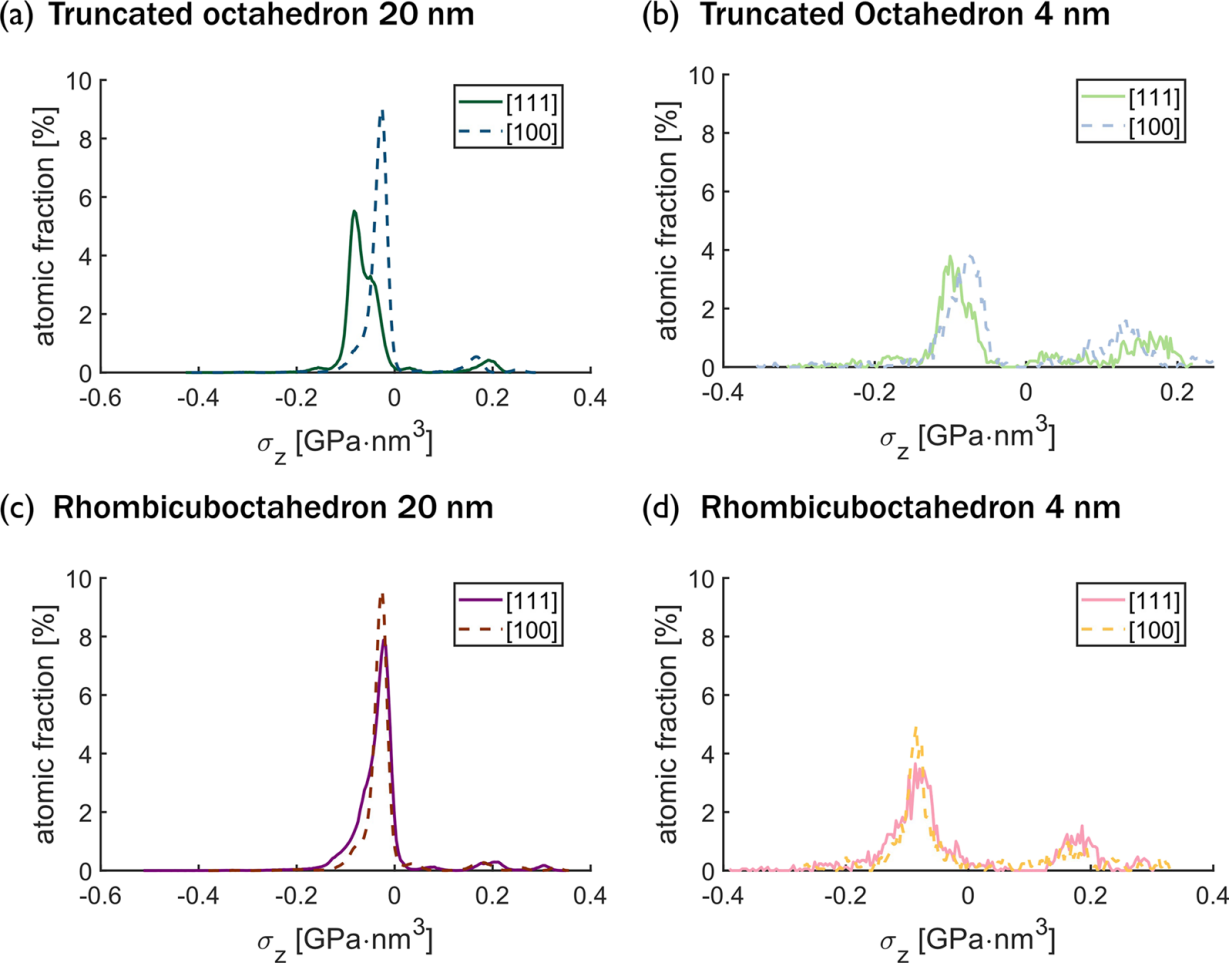
Distributions of the virial stress per atom for 20 nm and 4 nm nanoparticles. Virial stress histograms for atoms in cross-sections of thickness 7 nm parallel to the load direction for** a** 20-nm truncated octahedron, **b** 4-nm truncated octahedron,** c** 20-nm rhombicuboctahedron, and** d** 4-nm rhombicuboctahedron.

For the 20-nm nanoparticles loaded in the {111} orientation in Fig. 3a and c, the compressive stress is nearly homogeneous in the region between the top and bottom contact areas, subsequently called the core. The load is mostly supported by the core so the compressive stress is largest in that region. Toward the surface of the particles in the directions transverse to loading, the stress is lower and inhomogeneously distributed. The size of the core is related to the size of the contact area, so the homogeneous core-stress region is larger for the truncated octahedron than the rhombicuboctahedron. For the 20-nm particles, the core in the truncated octahedron accounts for nearly 42% of the atoms, while in the rhombicuboctahedron the core atoms are about 31%.

For the 20-nm nanoparticles compressed in the {100} orientation, Fig. 3b and d, the stress distribution is not homogeneous in the core region between the contact areas. For this loading direction, the nanoparticle is geometrically symmetric for planes parallel to the load, therefore the stress is also symmetric. The stress is lowest across the center area of the nanoparticles, and toward the regions outside the core.

In the 4-nm particles, the surface atoms are in tension and the highest compressive stress is observed near the contact areas, like in the 20-nm particles. The smaller particles also exhibit some of the same stress distribution patterns as the 20-nm particles, i.e., symmetry in the load direction for the {111} orientation and in the axes parallel to the load in the {100} orientation, although these features are less distinct in the 4-nm particles. For the truncated octahedron in both directions, the zone of largest compressive stress extends beyond the edges of the areas of contact, toward the diagonal facets. For the 4-nm rhombicuboctahedron in the {111} orientation, the compressive stress is largest adjacent to the contacts, but the high stress at the corners of the contacts is less predominant than in the 20-nm nanoparticle. For this same particle loaded in the {100} orientation, the stress is high and more homogeneous across the entire cross-section than for the 20-nm particle.

To quantify the distributions of stress in the nanoparticles, histograms of the per-atom stress at 3% strain, averaged over 2 ps, were calculated, as shown in Fig. 4. In all histograms, two peaks can be distinguished: a large peak corresponding to the negative, compressive stress and a small one for the positive, tensile stress. For the truncated octahedron, the compressive peak for {111} is to the left of the compressive peak for {100}, indicating that the atoms in the {111} orientation experience a higher compressive stress compared to the atoms of a nanoparticle compressed in the {100} orientation, as expected given the higher modulus of the {111} orientation. The key difference between the stress histograms for 4-nm and 20-nm particles is that the tensile stress peaks are larger in integrated intensity for the 4-nm particles than for the 20-nm nanoparticles. This confirms that a greater proportion of the atoms in the smaller particles is under tensile stress. Also, the compressive peak for the 4-nm particles is further left than the compressive peak for the 20-nm particles for both shapes and orientations, indicating a higher localized compressive stress in the 4-nm nanoparticles compared to the 20-nm particles. But, the peaks are wider for the 4-nm particles than the 20-nm particles, indicating that the stress is less homogeneously distributed in the 4-nm particles.

The inhomogeneous stress distributions in Fig. 3 and Fig. 4 give hints about the origins of the shape-, size-, and orientation-dependence of the effective elastic moduli calculated from all techniques in Fig. 2. The Hertz method assumes a specific stress distribution, which is not exhibited by these particles. The area-based methods yield an average stress, which cannot capture the wide variation with shape and orientation that is observed. Even the virial stress is effectively an average over the entire particle and so cannot capture the inhomogeneous stress distributions. These differences are exacerbated in smaller nanoparticles, which have a higher percentage of atoms in tension at the surface and regions of high compressive stress make up more of the particle than in large nanoparticles, leading to the observed size-dependence. The dependence of the bulk elastic modulus on orientation is well known, and explained by the positions of atoms in adjacent layers in the direction of loading. Specifically, in the {111} orientation, the atoms in adjacent layers are not stacked vertically and the distance between layers is $\sqrt{3}/3$ times the lattice whereas, in the {100} orientation, atoms are stacked vertically and the interplanar distance is half the lattice parameter (see Additional file 1: Fig. A4). For nanoparticles, these differences in atomic structure also affect the stress distribution. In the {111} orientation, the offset positions of atoms in adjacent layers enable load to be supported mostly by the core. In contrast, the stacked position of the atoms in the nanoparticles oriented in the {100} orientation causes a distribution of the forces in the lateral directions. For the effect of shape, the stress distributions suggest that the most significant factor is the size of the contact area which determines the size of the high-compressive-stress region at the top and bottom of the particle. The effect of nanoparticle shape is stronger for the {111} orientation because the different contact areas differ more between shapes and because the particles are not geometrically symmetric for axes parallel to the load in this orientation.

**Fig. 5:**
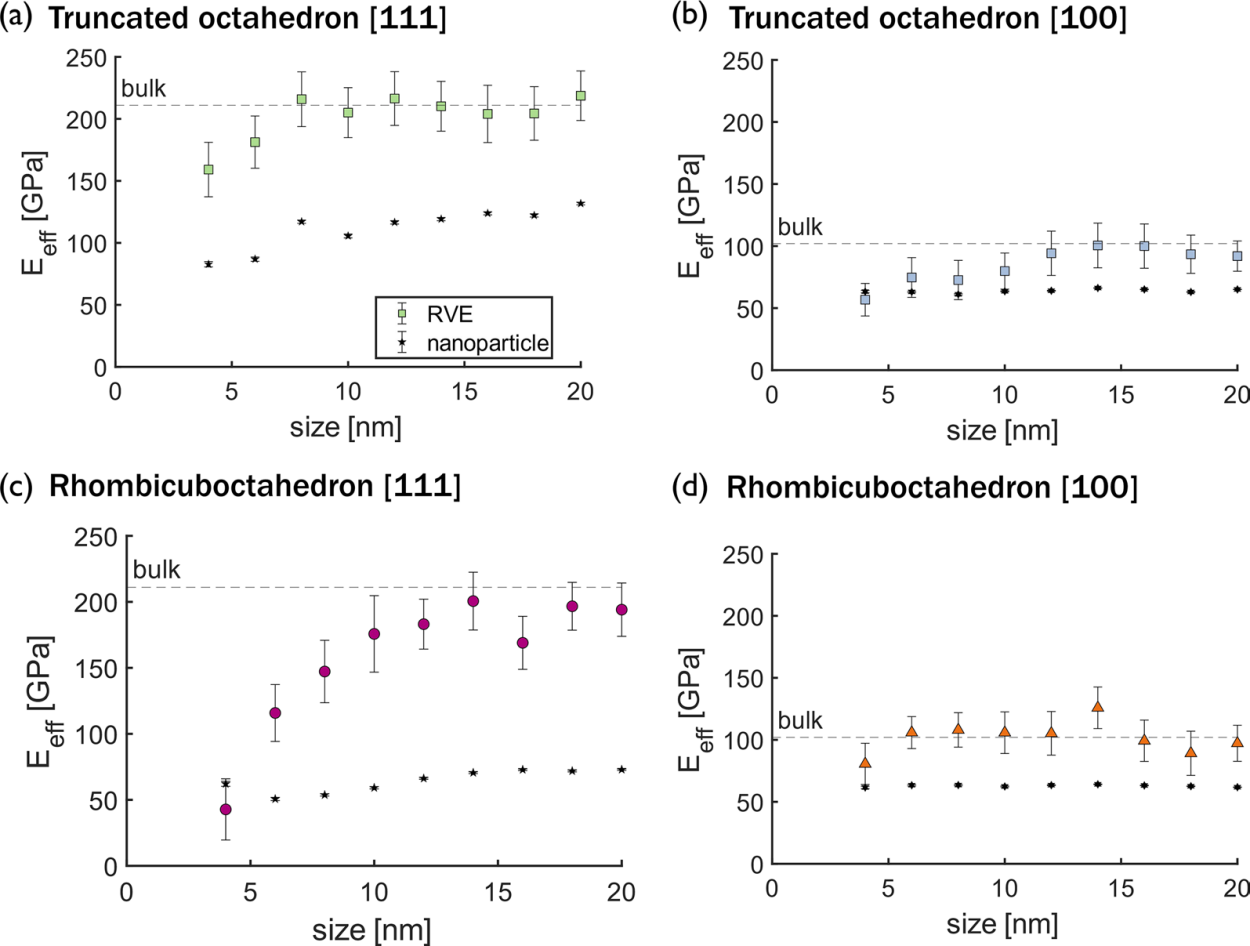
Effective elastic modulus versus nanoparticle size calculated for an RVE. Size and shape convergence of the effective elastic modulus calculated from the triaxial stress of an RVE at the center of the nanoparticles. Two shapes, (**a**, **b**) truncated octahedron and (**c**,** d**) rhombicuboctahedron, were teted in two crystallographic orientations with respect to the direction of the load, (**a**,** c**) correspond to the {111} orientation and (**b**,** d**) to the {100} orientation. The bulk values are shown as dashed lines and the effective elastic moduli calculated for the entire nanoparticles from the triaxial state are shown as solid stars

In all nanoparticles shown in Fig. 3, the stress in the center region of the nanoparticle is relatively homogeneous with respect to position, especially for the 20-nm particles. This is reasonable since the surface effects are smallest near the center of the nanoparticle. Based on this observation, the stress and strain of atoms in a representative volume element (RVE) at the center of the nanoparticle shown in (Additional file 1: Fig. A5) were calculated. The length of each side of the cubic RVE was 1.4 nm, five times the nearest-neighbor distance of platinum. Further, analysis of the stresses in the direction of the load and perpendicular to the direction of the load (Additional file 1: Fig. A6) showed that stress terms other than that in the loading direction are significant for these nanoparticles, especially for smaller sizes. This indicated that the typical unidirectional stress calculation may not fully capture nanoparticle elastic behavior.

Therefore, the effective elastic modulus of the RVE was calculated assuming the RVE in a triaxial state:

2 $$\begin{aligned} \frac{1}{E_{eff}}=\frac{\varepsilon _{3}}{\sigma _3{-\upsilon (\sigma _1{+\sigma _{2}})}} \end{aligned}$$

where $\sigma$ are stresses along the cube axes, $\varepsilon _{3}$ is strain in the direction of the load, and $\upsilon$ is the Poisson ratio for bulk platinum, equal to 0.385. The strain along the cube axes was calculated from the deformation of the cubic RVE, assuming that the element maintains planar opposite walls. The stress was obtained from the virial formulation normalized by the volume of the cube at each strain. The volume was calculated using a Delaunay triangulation; other volume calculations based on various definitions of atom radius were tested and the results were consistent. For the nanoparticles with facets aligned with {100} orientation, the cube directions lie along the orientation of the facets and the RVE was selected as a cube at the center of the nanoparticle. For the nanoparticles with {111} orientation, the nanoparticles were rotated in the plane normal to the direction of the load until one of the {111} facets aligned with the directions of the RVE cube. Because the angle between the {111} planes is 70$^{\circ }$, rather than the 90$^{\circ }$ angle of cube faces, this will introduce some error into the modulus calculation. However, the error is tolerated in order to create an algebraic equation to compute modulus.

Figure  5 shows the effective elastic modulus of the nanoparticles calculated using the RVE approach, compared to the effective elastic modulus calculated from the triaxial state for the entire nanoparticle (stars in the figure). Error bars reflect 95% confidence intervals. For all shapes and orientations, the effective elastic modulus is underestimated for the entire particle because the calculation includes the atoms near the surface that have lower compressive or even tensile stress (Fig. 3) such that the average stress is lower. This effect is mimimized by selecting an RVE near the center of the particle. To ensure that the RVE analysis does not depend on the interatomic potential, we repeated the compression simulations with a Tersoff potential [61] and compared the stress distributions in the particles to those shown in Fig. 3. Although the per-atom stress magnitudes differed between the two potentials, the stress distributions were similar. Most importantly, for both potentials, the stress was approximately homogeneous near the center of the particles. Using the RVE approach, the effective elastic modulus converges for larger particles to approximately the bulk value for a given orientation. For smaller particles, the effective elastic modulus of the RVE is smaller, consistent with previous analytical and numerical models [18, 36] and experimental studies [37, 38]. This is because the center of the nanoparticle will only exhibit bulk-like behavior if it is sufficiently far from the free surfaces; as the particle shrinks below a critical size, even the material at the center is affected by the free surface with its undercoordinated atoms and tensile stresses. This analysis demonstrates that, unlike conventional methods for extracting modulus from MD studies, the simple RVE approach can recover the bulk-like behavior and also differentiate the effects of nanoscale geometry from stress-distribution effects. Further, this analysis confirms that the effective elastic modulus is reduced with decreasing size, but that the critical size at which this reduction occurs depends on particle shape and loading orientation.

## Conclusions

MD simulations were used to investigate the effect of size, shape, and orientation on the elastic response of platinum nanoparticles to compression. First, commonly used methods for characterizing elastic modulus were compared and it was found that calculated values depended significantly on the methods used to calculate them. Also, for larger-size particles, none of the methods predicted convergence to the value calculated for bulk platinum in the same orientation. This limitation was explained by an analysis of the stress distributions in the nanoparticles, which revealed that the stresses in the particles were inhomogeneous and varied for different geometries, sizes, and orientations. The maximum compressive stress occurred near the contact with the indenter where the cross-sectional area was smallest, while the undercoordinated atoms near the free surface experienced tensile stress. For all particles, the stress became more homogeneous closer to the center of the nanoparticle where the surface effects were less significant. Therefore, we introduced a simple triaxial stress analysis of a representative volume element in the center of the nanoparticle as an alternative approach for calculating the effective elastic parameter of the particles. For large nanoparticles, the effective elastic modulus using an RVE matched the bulk value and was independent of particle shape. For smaller nanoparticles, the effective elastic modulus was lower than the bulk value because the free surfaces are inherently closer to the RVE, such that there is no longer any material in the particle that behaves in a truly bulk-like fashion. The RVE approximation presented here provides a consistent and physically meaningful measure of nanomaterial elasticity and also highlights the limitations of standard methods caused by the inhomogeneous distribution of stress in nanoparticles.

## Supplementary Information

**Additional file 1**. Effect of the simulation parameters of the virtual walls on elastic deformation, effect of the orientation in the deformation of fcc crystals, schematic of a representative element volume (RVE), and stresses in the direction-of and prependicular-to the load.

## Data Availability

The datasets used and/or analyzed during the current study are available from the corresponding author on reasonable request.

## Footnotes

1 Note that this use of “effective modulus” is not the same as in the Hertz model where it combines the properties of the sphere and substrate, but rather refers to the measured modulus of nanoparticles.
