# Supplementary material for: Atomistic Simulations of the Elastic Compression of Platinum Nanoparticles
Source: Nanoscale Res Lett. 2022 Oct 3;17:96. doi: 10.1186/s11671-022-03734-z (PMC9530103; doi:10.1186/s11671-022-03734-z)
Supplement: Supplementary file 1 — Additional file 1. Effect of the simulation parameters of the virtual walls on elastic deformation, effect of the orientation in the deformation of fcc crystals, schematic of a representative element volume (RVE), and stresses in the direction-of and prependicular-to the load. [file 11671_2022_3734_MOESM1_ESM.pdf]

Atomistic Simulations of the Elastic Compression of Platinum Nanoparticles - Supplementary Information

Ingrid M. Padilla Espinosa,[†] Tevis D.B. Jacobs,[‡] and Ashlie Martini[*,†]

†*Department of Mechanical Engineering, University of California, Merced, Merced, CA 95340, USA*

‡*Department of Mechanical Engineering and Materials Science, University of Pittsburgh, Pittsburgh, PA 15261, USA*

E-mail: amartini@ucmerced.edu

Phone: +1 209-228-2354

List of Figures

1 Force vs displacement for (a) different cutoff values and (b) different epsilon values. 3
2 Force vs displacement for different strain rates in s^{-1}. 4
3 Schematic of the plane of view of the cross-section selected for stress distribution analysis. 5
4 Atomic position of three consecutive planes in different orientations. 6
5 Schematic of the representative volume element of a truncated octahedron compressed in the {111} orientation and the {100} orientation 8

6 Stresses ($\sigma_x, \sigma_y, \sigma_z$) vs size for two shapes, (a,b) truncated octahedron and (c,d) rhombicuboctahedron, and two crystallographic orientations with respect to the direction of the load, (a,c) correspond to the {111} orientation and (b,d) to the {100} orientation. 9

Virtual indenter wall parameters: effect of cutoff and epsilon

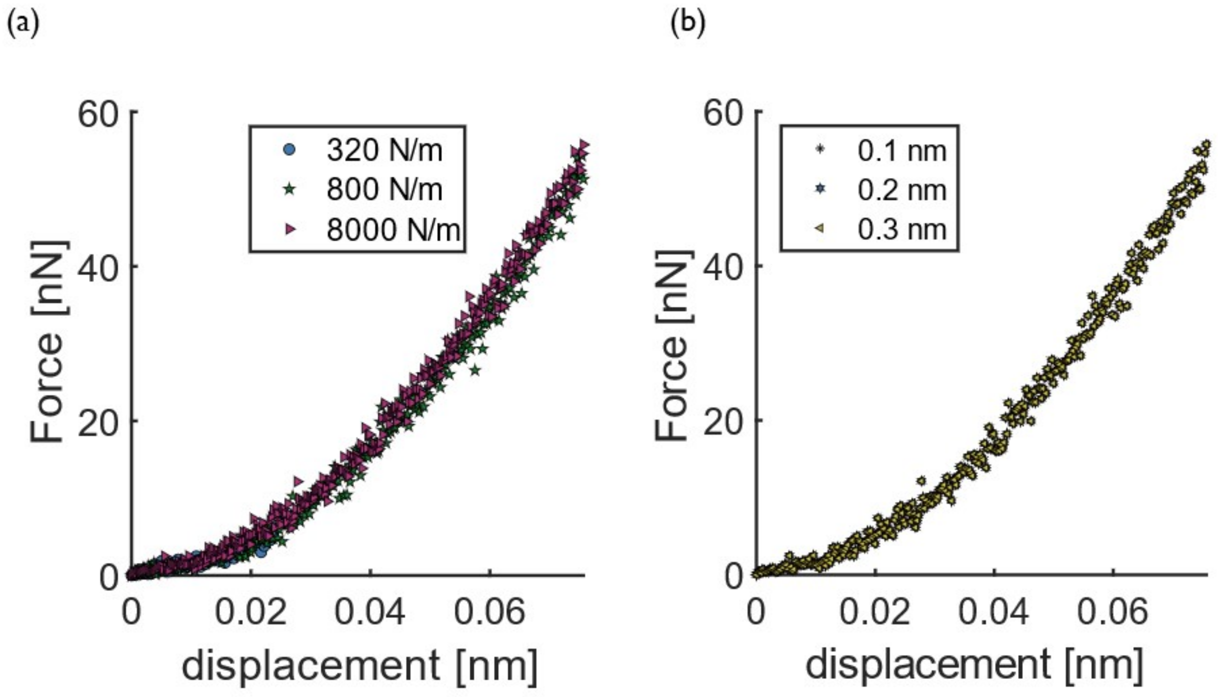

Figure A 1: Force vs displacement for (a) different cutoff values and (b) different epsilon values.

The spring constant (8000 N/m) and cutoff of 0.2 nm were selected for the simulations. An ideal spring constant of an indenter should be near infinite, so the elastic deformation occurs on the compressed material and not on the indenter tip. Then, an indenter is normally selected based on the stiffest material, following this, the highest epsilon value (8000 N/m) is used for all the compression tests. Although the cutoff value did not show an effect on the force vs displacement curve, a cutoff of 0.2 nm is used here. This value is larger than Pt atomic radius of 0.175 nm and smaller than Pt nearest neighbor 0.277 nm, Then the interaction forces between the virtual wall and the nanoparticle occurred at the atomic layer closest to the wall.

Effect of strain rate on the force vs displacement curve

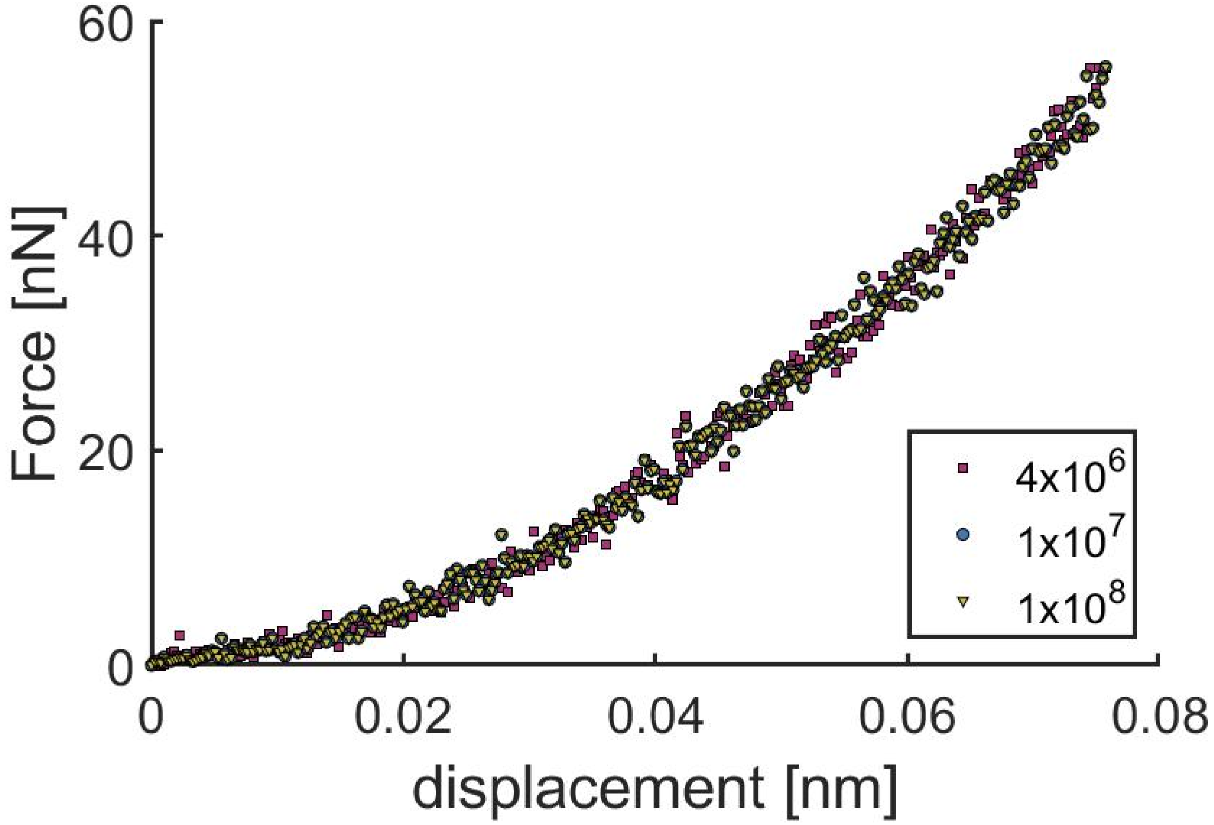

Figure A 2: Force vs displacement for different strain rates in s^{-1}.

Plane of view of the nanoparticle cross-section

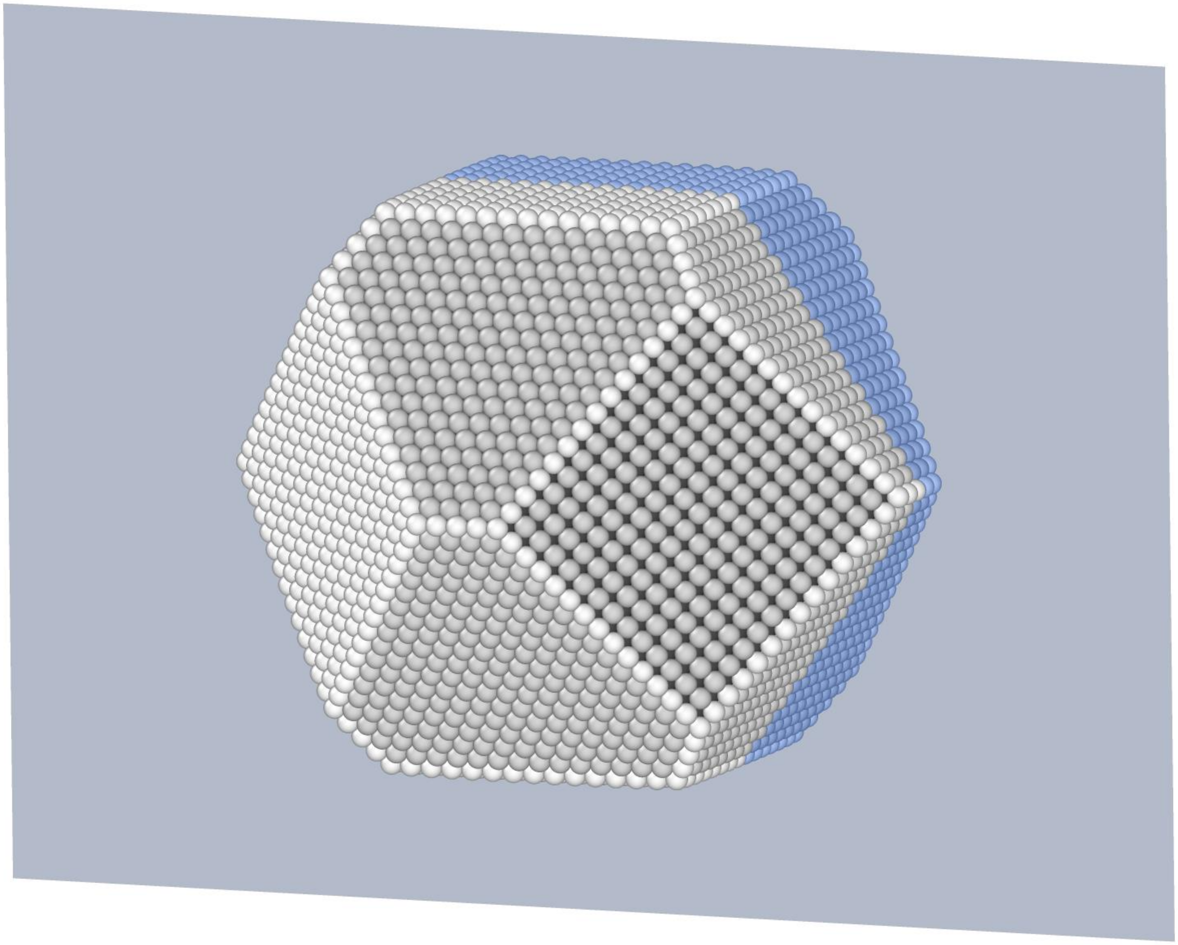

Figure A 3: Schematic of the plane of view of the cross-section selected for stress distribution analysis.

Interplanar positions and interactions of three planes along the load direction

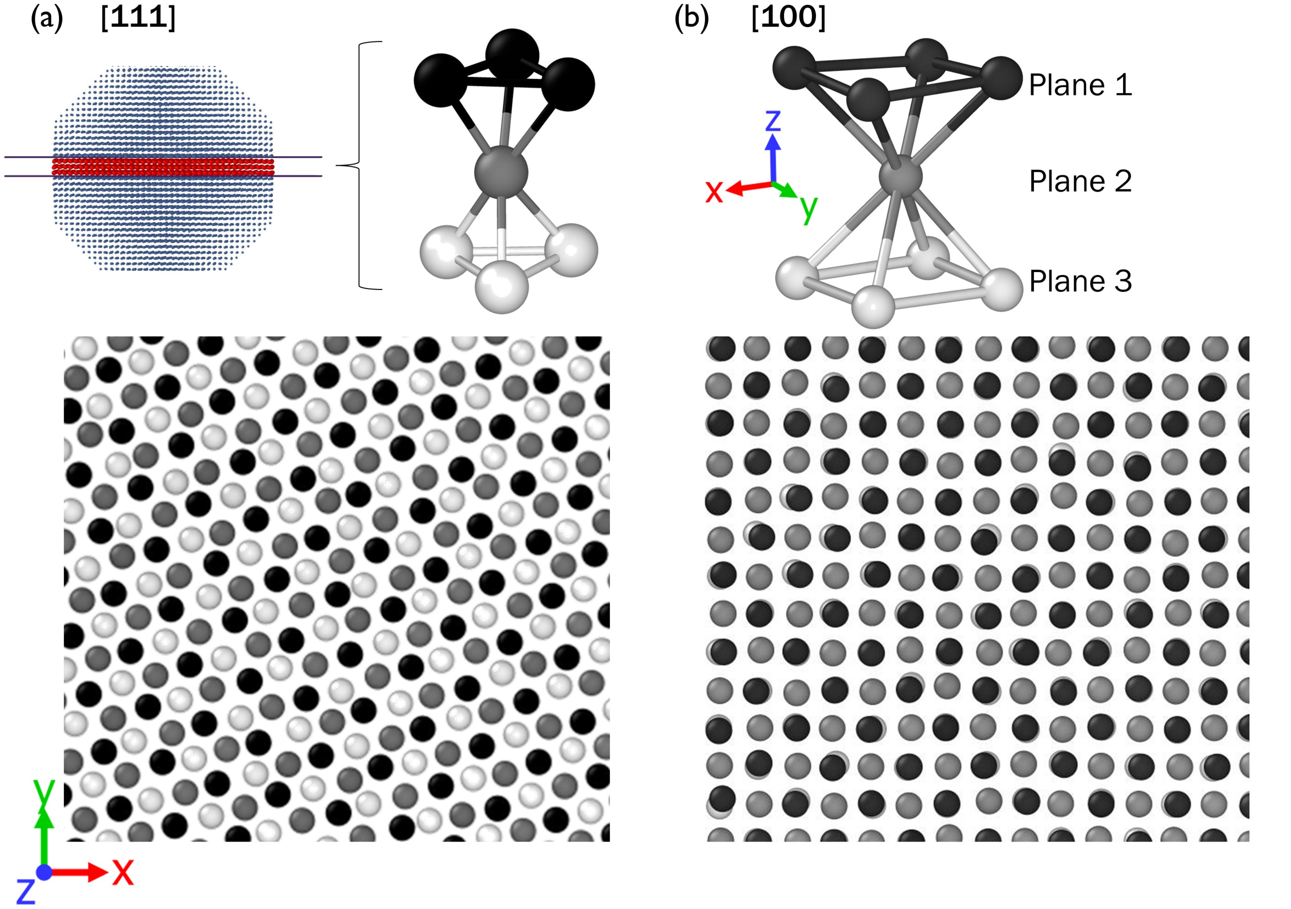

Figure A 4: Atomic position of three consecutive planes in different orientations.

Atomic position of three consecutive planes illustrated in (a), the top of the figure shows the interaction of an atom with the nearest atoms in the consecutive layer; the bottom figures show the atomic position from a view perpendicular to the planes for (a) {111} and (b) to {100} orientation. The color scale is darker for top layer (1) and lighter for bottom layer (3). This explains the orientation dependence of the effective elastic modulus: 211 GPa for the {111} orientation and 107 GPa for the {100} orientation for bulk platinum. For the {111} orientation, the atoms positions perpendicular to the load do not coincide with the position of the atoms

in the next two planes. Each one of the atoms interacts with three nearest atoms in the next consecutive layer, and the interplanar distance for FCC structures in the {111} orientation is $\sqrt{3}/3$ times the lattice. For the {100} orientation, the lateral position of the atoms coincides every other plane. Each atom interacts with four atoms in the next consecutive plane and the interplanar distance is half of the lattice parameter.

Representative Volume Element

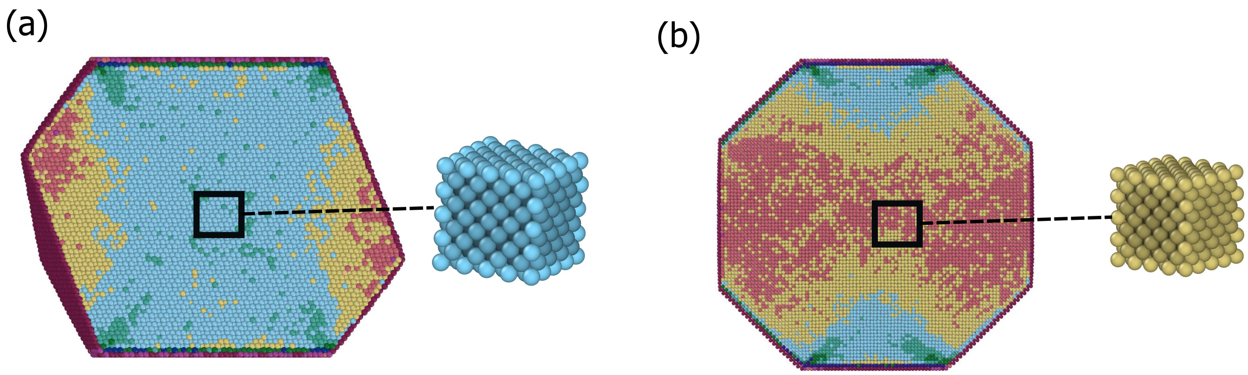

Figure A 5: Schematic of the representative volume element of a truncated octahedron compressed in the {111} orientation and the {100} orientation

Stresses at 2% strain in the direction of the load (σ_z) and perpendicular to the direction of the load (σ_x and σ_y)

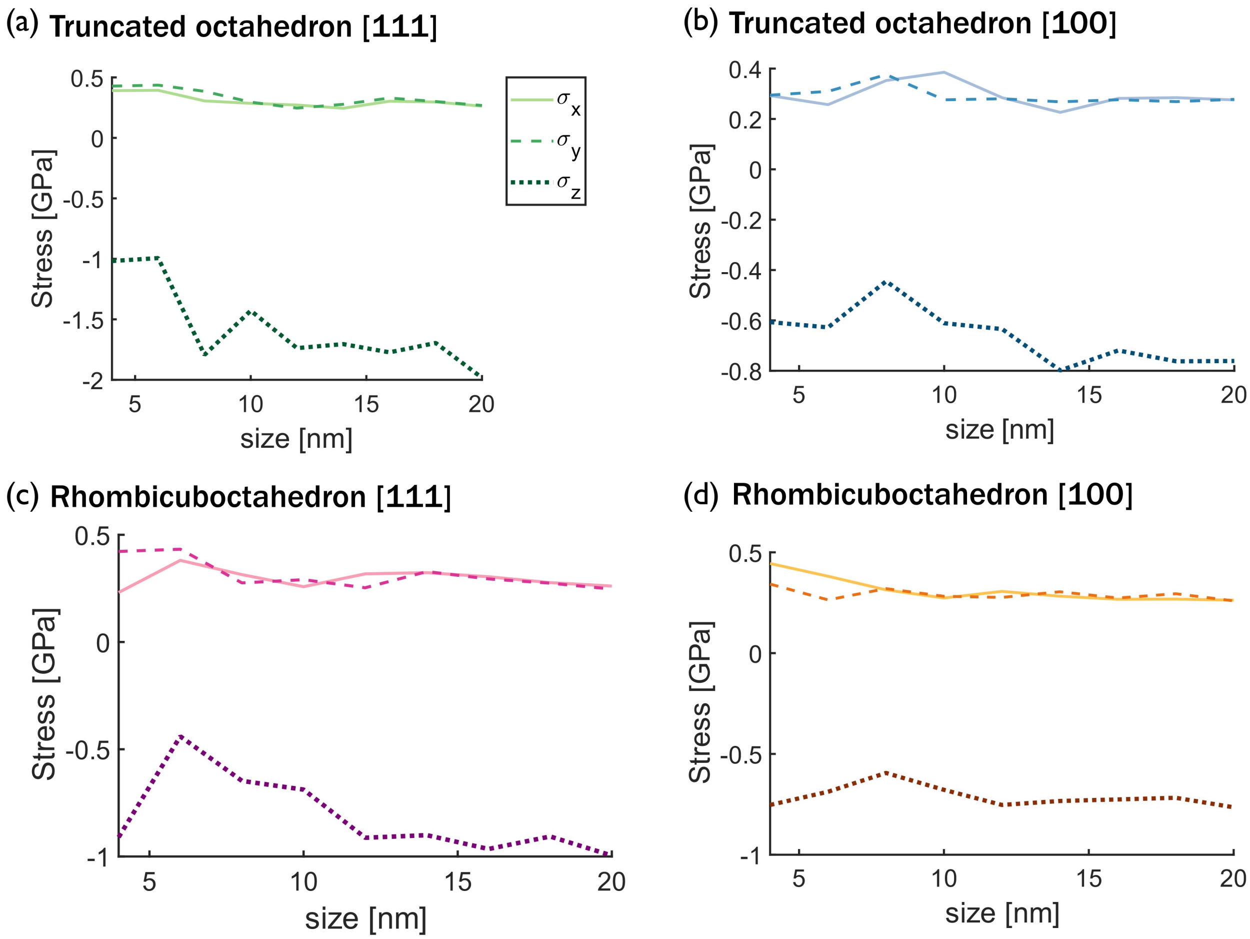

Figure A 6: Stresses ($\sigma_x, \sigma_y, \sigma_z$) vs size for two shapes, (a,b) truncated octahedron and (c,d) rhombicuboctahedron, and two crystallographic orientations with respect to the direction of the load, (a,c) correspond to the {111} orientation and (b,d) to the {100} orientation.
